# 

**Published:** 1878-07

**Authors:** 


### CONTENTS:

ARTICLE I.—The Proximate Causes of Decay of the Teeth of Americans. By Prof. Hodgkin.......... 97
ARTICLE II.—Dental Decay and Filling Materials Considered in their Electrical Relations. By S. B. Palmer, D. D S.......... 105
ARTICLE III.—The Metric System. By Prof. J. B. Hodgkin.......... 111
ARTICLE IV.—The Influence of Cheeks and Tongue and Lips on Gums and Jaws. By C. Sauer.......... 116
ARTICLE V.—Restoration of Inferior Maxilla after removal. By J. B. Hodgkin, D. D. S 122
ARTICLE VI.—Requisite Knowledge for the Practice of Dentistry. By Frank M. Odell, D. D. S., M. D.......... 126
ARTICLE VII.—Anæsthesia by Rapid Respiration. By Dr. A. H. R. Guiley.......... 128
ARTICLE VIII.—Pennsylvania State Dental Society.......... 130
ARTICLE IX —Cheiloplasty. By John H. Brinton M. D.......... 132
ARTICLE X.—Treatment of Stomatitis Materna. By E. P. Hurd, M. D.......... 134
ARTICLE XI.—Local Effects of Astringents upon the Blood Vessels. By Dr. A. Rosenstein.......... 135

### EDITORIAL, ETC.

Hope for the Dental Profession. The Cummings' Patent.......... 137
Chloroform and the Dentists.......... 140
College Changes.......... 143

### BIBLIOGRAPHICAL.

Proceedings of Southern and American Dental Conventions.......... 143

